# Acetylcholine From Tuft Cells: The Updated Insights Beyond Its Immune and Chemosensory Functions

**DOI:** 10.3389/fcell.2020.00606

**Published:** 2020-07-07

**Authors:** Jun Pan, Leyi Zhang, Xuan Shao, Jian Huang

**Affiliations:** ^1^Key Laboratory of Tumor Microenvironment and Immune Therapy of Zhejiang Province, Second Affiliated Hospital, Zhejiang University School of Medicine, Hangzhou, China; ^2^Cancer Institute (Key Laboratory of Cancer Prevention and Intervention, National Ministry of Education), Second Affiliated Hospital, Zhejiang University School of Medicine, Hangzhou, China; ^3^Department of Breast Surgery, Second Affiliated Hospital, Zhejiang University School of Medicine, Hangzhou, China

**Keywords:** acetylcholine, tuft cells, non-neuronal cholinergic system, neuroendocrine, cell biology

## Abstract

Tuft cells, rare solitary chemosensory cells, are distributed in mucosal epithelium throughout mammalian organs. Their nomenclatures are various in different organs and may be confused with other similar cells. Current studies mainly focus on their chemosensory ability and immune functions in type 2 inflammation. Several state-of-the-art reviews have already systematically discussed their role in immune responses. However, given that tuft cells are one of the crucial components of non-neuronal cholinergic system, the functions of tuft cell derived acetylcholine (ACh) and the underlying mechanisms remain intricate. Existing evidence demonstrated that tuft cell derived ACh participates in maintaining epithelial homeostasis, modulating airway remodeling, regulating reflexes, promoting muscle constriction, inducing neurogenic inflammation, initiating carcinogenesis and producing ATP. In this review, the ACh biosynthesis pathways and potential clinical applications of tuft cells have been proposed. More importantly, the main pathophysiological roles and the underlying mechanisms of tuft cell derived ACh are summarized and discussed.

## Introduction

The definition of non-neuronal acetylcholine (ACh) was originally proposed by Morris in 1966, who demonstrated the synthesis of ACh in placenta (Morris, 1966). Since then, the expressions of components involved in cholinergic system, including choline acetyltransferase (ChAT), vesicular ACh transporter (VAChT), and high-affinity choline transporter (CHT1), have been confirmed in T cells (Kawashima et al., 1989; Rinner and Schauenstein, 1993; Rosas-Ballina et al., 2011; Dhawan et al., 2016), B cells (Reardon et al., 2013), NK cells (Jiang et al., 2017), dendritic cells (Reardon et al., 2013), macrophages (Reardon et al., 2013), endothelial cells (Parnavelas et al., 1985), and epithelial cells (Moffatt et al., 2004; Proskocil et al., 2004; Kummer et al., 2008; Wolf-Johnston et al., 2012; Bader et al., 2014; Kummer and Krasteva-Christ, 2014; Bankova et al., 2018).

Tuft cells, also termed as brush cells, microvillus cells and chemosensory cells, were firstly demonstrated in rat tracheal mucosal epithelium in 1956 (Rhodin and Dalhamn, 1956). Although they have been discovered for more than 60 years, their functions remain elusive. In previous studies, tuft cells have been proven to participate in chemosensing (Krasteva et al., 2012a; Deckmann et al., 2014), mediating neurogenic inflammation (Saunders et al., 2014), expressing Th2-related genes (Bezencon et al., 2008) and producing neurotransmitter ACh (Krasteva et al., 2011, 2012b). Not until three studies published in 2016 did researchers attach importance to the immunomodulatory effects of tuft cells (Gerbe et al., 2016; Howitt et al., 2016; von Moltke et al., 2016). But the function of non-neuronal ACh synthesis of tuft cells has been neglected for decades. Recently, Schütz et al. (2019) identified the distribution and molecular signatures of cholinergic tuft cells in human digestive tract, including gastrointestinal, biliary and pancreatic systems. Given that tuft cell is the only epithelial cellular source of ACh in human digestive tract (Schütz et al., 2019), tuft cell-derived cholinergic signaling may play a considerable role and should be paid more attention. In the light of this rationale, this review, summarizing older reviews and novel advances, focuses on the pathophysiological roles of tuft cell derived ACh.

## Characteristics of Tuft Cells

### Definition

Lacking consensus on their functions, tuft cells were identified by their morphology in the past. They are columnar or flask-like in shape with ‘tuft-like’ brush of apical microvilli (500–1000 nm in length and 150–180 nm in width) extended into the hollow lumen (except thymus) (Reid et al., 2005; O’Leary et al., 2019). Tuft cells has been defined as an epithelial lineage from four aspects (Schneider et al., 2019). The first aspect remained the morphological criteria described above. The second term was the transcription factor POU domain, class 2, transcription factor 3 (POU2F3), which had been proven as a master regulator for the generation and functions of tuft cells in multiple tissues including trachea, thymus, urethra, stomach, auditory tube, pancreatic duct, and large intestine (Gerbe et al., 2016; Yamashita et al., 2017; Huang et al., 2018). The third requirement was maintaining components in taste-signaling. Most of them expressed G-protein α-gustducin (GNAT3), phospholipase Cβ2 (PLCβ2) and transient receptor potential cation channel subfamily M member 5 (TRPM5) (Kaske et al., 2007; Bezencon et al., 2008). The last aspect was the expressions of effector molecules, including IL-25 and components of eicosanoids biosynthesis pathway, such as cyclooxygenase 1 and 2 (COX1, COX2) (Bankova et al., 2018). However, they also declared that the consensus of tuft cells had not been reached by the research community. Besides the core gene signatures, ACh is one of the additional soluble effector molecules biosynthesized by tuft cells. Current functional studies of tuft cells have laid particular emphasis on IL-25 and ACh (Schneider et al., 2019).

Though having been discovered for a long time, “tuft cell” is a recently-emerged term. The nomenclatures of tuft cells are diverse, such as brush cells (respiratory tract), caveolated cells (gastrointestinal tract), microvillus cells (nasopharynx), fibrillovesicular cells (forestomach and glandular stomach), solitary chemosensory cells (SCCs) (respiratory tract), etc.

### Distribution and Markers

Although tuft cells were firstly discovered in rat trachea, they have been well described in most mucosal epithelia. In rodents, they have been found through respiratory system (nasal cavity Finger et al., 2003; Ogura et al., 2010, and trachea Krasteva et al., 2011; Saunders et al., 2013) to the digestive system (salivary glands (Sato and Miyoshi, 1988), gastrointestinal tract (Bezencon et al., 2008; Schütz et al., 2015; Hayakawa et al., 2017; Konishi et al., 2019), biliary tract (Schütz et al., 2015) and colon (Silva, 1966; Howitt et al., 2016) and the urogenital tract (Deckmann et al., 2015), as well as the thymus (Panneck et al., 2014), auditory tube (Krasteva et al., 2012b), and taste buds (Sukumaran et al., 2017).

However, there are variations between tuft cells in different species as well as different organs in individual. In the respiratory tract, tuft cells have been observed throughout the respiratory tract in rat but were nearly absent in the intrapulmonary airways below the bronchial branch point in mice. The discrepancy also occurs in the biliary tract. ChAT^+^ cholinergic tuft cells have been found in extra-hepatic peribiliary glands but are absent in gall bladder and common bile duct epithelium in human (Schütz et al., 2019), whereas they scatter throughout the gall bladder and extra-hepatic bile duct in rodents (Schütz et al., 2015). It’s not confusing that tuft cells should not be defined into one single lineage because of the multiple characteristics and wide dispersal throughout mucosal surfaces. It has been reported that the expression of ChAT can be detected in most tuft cells, but the expression of VAChT is not common to all tuft cells situated in the mucosa of mouse gastrointestinal and biliary tract (Schütz et al., 2015). Although tuft cells in mice trachea and intestine maintain the same unique morphology and transcriptional signature (Bankova et al., 2018; Bouchery and Marsland, 2018), the results of RNA sequencing (Montoro et al., 2018; Nadjsombati et al., 2018; Plasschaert et al., 2018) and other related studies have reconfirmed that tuft cells in different microenvironment evolved different expressions of surface markers. In addition to POU2F3 and TRPM5 mentioned above, researchers also found increasing lineage markers of tuft cells, including COX1, COX2, leucine rich repeat containing G protein-coupled receptor 5 (LGR5), SRY-Box transcription factor 9 (SOX9), achaete-scute family BHLH transcription factor 2 (ASCL2), HOP homeobox (HOPX), p-EGFR, growth factor independent 1B (GFI1b), CHAT, cytokeratin 18 (CK18), villin, Advillin (AVIL), etc. (Haber et al., 2017; McKinley et al., 2017; Huang et al., 2018; Schütz et al., 2019; Esmaeilniakooshkghazi et al., 2020). The representative markers of tuft cells from different species and different organs are summarized in Table 1. Besides the divergences of marker expressing on tuft cells in different microenvironment, heterogeneity exists, therefore tuft cells can be distinguished into subtypes. In murine airway, tuft cells can be partitioned into three clusters: immature tuft cells, tuft-1cells (express taste transduction genes) and tuft-2 cells (express leukotriene biosynthesis genes) (Montoro et al., 2018). But in murine small intestine, by using transcriptomics, Haber et al. suggested that tuft cells divided into two subtypes: tuft-1 cells associated with neuromodulation, while tuft-2 cells involved more in immunological functions (Haber et al., 2017).

**TABLE 1:** Markers and main functions of tuft cells from different organs.

**Organ**	**Species**	**Markers**	**Main functions**	**Additional characteristics**
Respiratory tract	Rodents	DCLK1, POU2F3, CK18, Villin, Fimbrin, AVIL, SOX9, ChAT, VAChT, GNAT3, PLCβ2, TRPM5, TAS2R108, GNG13, ALOX5AP, IL-25, IL-17RB (Yamashita et al., 2017; Montoro et al., 2018; Plasschaert et al., 2018; Rane et al., 2019; Hollenhorst et al., 2020).	1. Participate in type 2 immune response (Miller et al., 2020) and neurogenic inflammation (Kummer and Krasteva-Christ, 2014; Snelgrove and Lloyd, 2018). 2. Generate CysLT in response to local damage (Ualiyeva et al., 2020). 3. Sense taste signals and quorum sensing molecules (Krasteva et al., 2012a) and regulate mucociliary clearance (Krasteva et al., 2012a; Hollenhorst et al., 2020; Perniss et al., 2020). 4. Regulate respiratory reflexes (Krasteva et al., 2011). 5. Maintain epithelial homeostasis (Kummer and Krasteva-Christ, 2014) and regulate airway remodeling (Rane et al., 2019). 6. Progenitor of a subset of SCLC (Huang et al., 2018).	Solitary entities.
	Human	POU2F3, SOX9, ChAT, TRPM5, GF11B, ASCL2, AVIL (Huang et al., 2018).		
Auditory tube	Rodents	DCLK1, POU2F3 (Yamashita et al., 2017), ChAT, VAChT, CHT1, GNAT3, PLCβ2, TRPM5 (Krasteva et al., 2012b).	Sense the composition of the luminal microenvironment and deliver signals to CNS via attached sensory nerve fibers (Krasteva et al., 2012b).	Solitary entities.
Taste buds	Rodents	DCLK1, Advillin (Schütz et al., 2019), ChAT, VAChT, CHT1, GNAT3, PLCβ2, TRPM5 (Dando and Roper, 2012).	1. Form an autocrine feedback of ACh and potentiate taste-evoked signals (Sukumaran et al., 2017). 2. Relase ATP (Dando and Roper, 2012).	Type II taste cells.
Thymus	Rodents	DCLK1, POU2F3 (Yamashita et al., 2017), TRPM5, GNAT3, PLCβ2, TAS2R, ChAT, LRMP, AVIL, GNG13, L1CAM, SOX9 (Bornstein et al., 2018).	Promote a microenvironment enriched in IL-4 and regulate the development and polarization of thymic invariant natural killer T cells (Bornstein et al., 2018; Miller et al., 2018).	Non-random distributed throughout the medulla (Miller et al., 2018).
Stomach	Rodents	DCLK1 (Saqui-Salces et al., 2011), POU2F3 (Yamashita et al., 2017), ChAT (lacking VAChT and CHT1), GNAT3, PLCβ2, TRPM5, COX1, COX2, HPGDS, CK18 (Schütz et al., 2015).	1. Sense taste signals and defense harmful substances (Schütz et al., 2015). 2. Expand in hyperplasia (Saqui-Salces et al., 2011) and participate in tumor initiation (Hayakawa et al., 2017).	Gathered in gastric groove (Schütz et al., 2015).
	Human	Lack cholinergic tuft cells (Schütz et al., 2019).		
Small intestine	Rodents	DCLK1, POU2F3 (Yamashita et al., 2017), CK18, Fimbrin, Advillin (but not villin) (Schütz et al., 2019; Esmaeilniakooshkghazi et al., 2020), ChAT (lacking VAChT and CHT1) (Schütz et al., 2015), COX1, COX2, HPGDS, LGR5 SOX9 (Gerbe et al., 2011), SUCNR1 (Nadjsombati et al., 2018), IL-25, TSLP, IL4RA, IL13RA1, IL17RB (Haber et al., 2017), Hopx, p-EGFR (McKinley et al., 2017).	1. Participate in type 2 immune response to against helminth, protists and virus infection (Gerbe et al., 2016; von Moltke et al., 2016; Nadjsombati et al., 2018; Wilen et al., 2018). 2. Maintain the epithelial homeostasis (Middelhoff et al., 2020).	Solitary entities.
	Human	CK18, Villin, Advillin, ChAT, COX1, COX2, p-EGFR, HPGDS, FLAP (McKinley et al., 2017; Schütz et al., 2019).		
Large intestine	Rodents	DCLK1, POU2F3 (Yamashita et al., 2017), CK18, Villin, Fimbrin, ChAT, VAChT (except VAChT^+^ tuft cells in ascending colon), lacking CHT1 (Schütz et al., 2015), COX1, COX2, HPGDS, COX2, p-EGFR (Schütz et al., 2015; McKinley et al., 2017).	1. Have the potential to initiate tumor (Westphalen et al., 2014) and be an underlying tracing target (Goto et al., 2019). 2. A probable marker for Hirschesprung’s Disease (O’Donnell et al., 2020). 3. Protect body from potential hazardous compounds (Yamashita et al., 2017).	Scatter throughout the epithelial sheet but enrich in the villar region in the ileum (Kaske et al., 2007).
	Human	CK18, Advillin, ChAT, IL17RB, COX2, p-EGFR (McKinley et al., 2017; Goto et al., 2019; Schütz et al., 2019).		
Pancreatic ducts	Rodents	DCLK1 (not specific to tuft cells) (DelGiorno et al., 2020), POU2F3, TRPM5, lacking SUCNR1 (Yamashita et al., 2017).	Secrete IL-25 to promote epithelial recovery and inhibit the initiation of pancreatic ductal adenocarcinoma (DelGiorno et al., 2020).	Solitary entities (Schütz et al., 2015). Absent under normal condition, but appeared under inflammation and tumor initiation (DelGiorno et al., 2020).
	Human	CK18, Villin, Advillin, ChAT, COX1, HPGDS, FLAP (Schütz et al., 2019).		
Biliary tract	Rodents	DCLK1, POU2F3 (Yamashita et al., 2017), ChAT (lacking VAChT and CHT1), GNAT3, PLCβ2, TRPM5, COX1, COX2, HPGDS, CK18 (Schütz et al., 2015).	Sense taste signals (Schütz et al., 2015).	Solitary entities (Schütz et al., 2015)
	Human	CK18, Villin, ChAT, COX1, HPGDS (Schütz et al., 2019).		
Urethra	Rodents	POU2F3 (Yamashita et al., 2017), Villin (Deckmann et al., 2015), ChAT (lacking VAChT and CHT1) (Schütz et al., 2015), GNAT3, PLCβ2, TRPM5 (Deckmann et al., 2014).	Respond to taste signals as sentinels of urinary tract and cause constriction of bladder detrusor muscles (Deckmann et al., 2014).	Solitary entities (Schütz et al., 2015).
	Human	Villin (Deckmann et al., 2015), ChAT, GNAT3, PLCβ2, TRPM5 (Deckmann et al., 2014).		
	Cynomolgus	Villin, ChAT, GNAT3 (expressed weakly), PLCβ2, TRPM5 (Deckmann et al., 2015).		
	Marmoset	Villin, ChAT, GNAT3, PLCβ2 (expressed weakly), TRPM5 (expressed weakly) (Deckmann et al., 2015).		
	Dog	Villin, ChAT, GNAT3 (expressed weakly), PLCβ2 (expressed weakly), TRPM5 (Deckmann et al., 2015).		
	Badger	Villin, ChAT, GNAT3 (expressed weakly), PLCβ2 (expressed weakly), TRPM5 (Deckmann et al., 2015).		
	Cat	Villin (in 1/3 samples), GNAT3, PLCβ2 (in 1/3 samples), TRPM5 (Deckmann et al., 2015).		
	Cattle	Villin (in 3/5 samples), ChAT (uncertain), GNAT3, PLCβ2 (in 1/5 samples), TRPM5 (in 3/5 samples) (Deckmann et al., 2015).		
	Red deer	Villin (in 1/2 samples), ChAT (in 1/2 samples), GNAT3, PLCβ2, TRPM5 (Deckmann et al., 2015).		
	Pigs	Villin (in 2/3 samples), ChAT, GNAT3 (in 2/3 samples), PLCβ2 (in 2/3 samples), TRPM5 (in 2/3 samples) (Deckmann et al., 2015).		
	Horse	Villin (in 1/2 samples), GNAT3, PLCβ2 (in 1/2 samples), TRPM5 (Deckmann et al., 2015).		

*GNG13, Guanine nucleotide−binding protein subunit gamma−13; HPGDS, Hematopoietic prostaglandin-D; mTEC, medullary thymic epithelial cells; TSLP, Thymic stromal lymphopoietin.*

Recently, cholinergic tuft cells have been identified throughout human alimentary tract (Schütz et al., 2019). In contrast with tuft cells in rodents, cholinergic tuft cells were absent in human stomach, gall bladder, common duct of biliary tract and main pancreatic duct. However, they can be found in the villi and crypts in the small and large intestine, the epithelia of extra-hepatic peribiliary glands, the epithelia of small and medium-size intra- and inter-lobular pancreas ducts (Schütz et al., 2019).

## Acetylcholine Biosynthesis and Release in Tuft Cells

**FIGURE 1:**
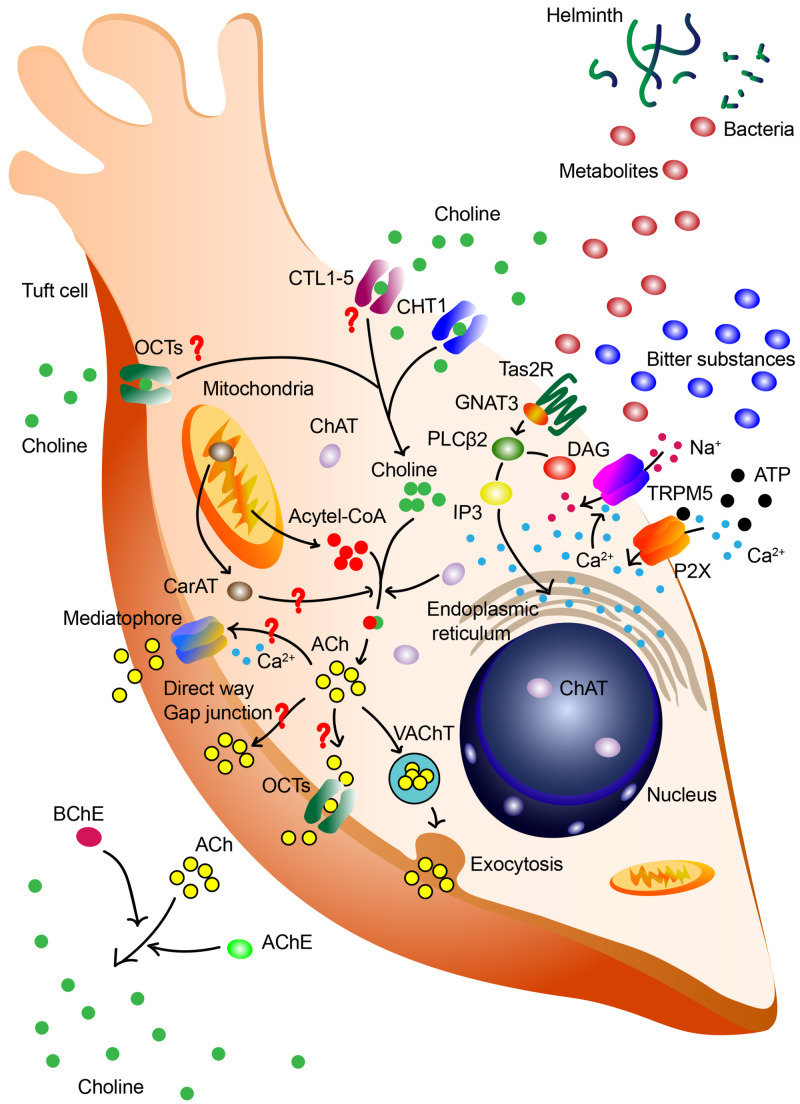
Synthesis and release of ACh by tuft cells. Tuft cells may synthesize and release ACh through canonical biosynthesis pathway and other different pathways. Various signals, including bitter substances, ATP and bacterial metabolites, are capable of triggering this process. ATP increases intracellular Ca^2+^ level via purinergic receptor P2X and the acceleration of Ca^2+^ promotes the lease of ACh. Bitter substances and bacterial metabolites are sensed by taste receptor type 2 (Tas2R), influence downstream G-protein α-gustducin (GNAT3) and phospholipase C β2 (PLCβ2), induce perturbation of intracellular Ca^2+^, activate TRPM5 and subsequently result in the release of ACh. In the canonical ACh biosynthesis pathway, extracellular choline is imported via CHT1, catalyzed into ACh with mitochondria-derived acetyl-CoA by ChAT in the cytoplasm. The ACh is then packaged into vesicles and released via VAChT in the exocytosis manner. Redundant ACh will be degraded into choline via acetylcholinesterase (AChE) and butyrylcholinesterase (BChE), and this extracellular choline may be re-uptake for the next cycle. However, there are still questions in the ACh biosynthesis in tuft cells. It is deduced that the synthesis of ACh may also be catalyzed by mitochondrial enzyme CarAT. In gastrointestinal and biliary tract, tuft cells lack the expression of VAChT and CHT1. These cells are speculated to re-uptake choline via CTL1-5 and OCTs, release ACh via direct way, OCT1/2-mediated way, and proteolipid mediatophore or even gap junction.

The biosynthesis of ACh has been well established in neurons. In brief, extracellular choline is imported via CHT1, catalyzed into ACh with mitochondria-derived acetyl-CoA by ChAT in the cytoplasm. The ACh is then packaged into vesicles and released via VAChT. However, the biosynthesis and release of ACh in tuft cells remains largely unknown. So far, only the expression of ChAT on tuft cells, and the release of ACh from tuft cells have been experimentally validated (Ting and von Moltke, 2019; Hollenhorst et al., 2020). On account of the absence of VAChT and CHT1 in gastrointestinal tuft cells, we highly deduce that, except the canonical biosynthesis pathway of ACh, tuft cells may synthesize and release ACh through different pathways (Figure 1).

### Signals Triggering ACh Biosynthesis and Release in Tuft Cells

Various signals have the ability to trigger the release of ACh from tuft cells, including cigarette smoke, bitter, ATP and bacterial metabolites (Montalbano et al., 2014; Saunders et al., 2014; Koarai and Ichinose, 2018; Ualiyeva et al., 2020). Previous study in mice demonstrated that ATP increased intracellular Ca^2+^ level in TRPM5^+^ microvillus cells and the number of responding cells in main olfactory epithelium (Fu et al., 2018). This up-regulation of Ca^2+^ level was mediated by purinergic receptor P2X in a dose-dependent manner. The oscillation of elevated Ca^2+^ levels subsequently promotes the release of ACh (Fu et al., 2018). However, bitter and other diverse substances, whether noxious or innocuous, trigger ACh biosynthesis of tuft cells via canonical taste transduction cascade depended on gustducin and TRPM5 (Saunders et al., 2014; Hollenhorst et al., 2020). This phenomenon could only be seen in the nasal epithelium. The inhibition of the taste transduction signaling abolished the elevated Ca^2+^ levels and subsequent ACh-release in mice (Hollenhorst et al., 2020). On the contrary, the activation of taste transduction increased the release of tuft cells derived ACh, and this neurotransmitter interacted with receptors on nerve fiber terminal to evoke nerve fibers-related inflammation and trigger protective reflexes (Krasteva et al., 2011). The blockage of nicotinic, not muscarinic, receptors prominently suppressed this inflammatory process. Genetic deletion of gustducin or TRPM5 mice revealed a defect in neurogenic inflammation triggered by the prototypical bitter compound denatonium. Diverse blocking experiments credibly suggested the link between taste transduction and cholinergic signaling in SCCs (Saunders et al., 2014).

### Transport of Choline

Conventional procedure of choline re-uptake is mediated by CHT1, a rate-limiting protein encoded by *Slac5a7*. CHT1 is a high-affinity choline transport system belonging to the Na^+^-dependent glucose transporter family (Okuda et al., 2000). Several studies have verified the expression of CHT1 in non-neuronal cells in bronchial epithelia cells (Proskocil et al., 2004), tracheal epithelium (Pfeil et al., 2003) and colon (Bader et al., 2014), indicating the role of CHT1 in choline re-uptake in these cells. However, the expression of CHT1 has not yet been found on tuft cells. Using *in situ* hybridization and immunohistochemistry, Schütz et al. (2015) verified that CHAT^+^ brush cells in mice gastrointestinal and biliary tract lacked the expression of CHT1. This absence of CHT1 in non-neuronal ACh-synthesis cells has also been proven in rat colon epithelium (Yajima et al., 2011). Apart from CHT1, choline transporter-like proteins 1–5 (CTL1-5) have been demonstrated to participate in choline re-uptake with an intermediate-affinity (Yajima et al., 2011). And the existences of CTL1-5 have been described in epithelium rather than specifically in tuft cells. Organic cation transporters (OCT) consist of three subtypes called OCT1, OCT2, and OCT3. They are plasma membrane transporters playing important roles in uptake and excretion. In human pulmonary respiratory epithelium, it is demonstrated that human OCT1 (hOCT1) and hOCT2 participate in the uptake of choline, mediating the non-neuronal autocrine and paracrine cholinergic regulation (Koepsell et al., 2007). It’s still nothing but a conjecture that tuft cells express and rely on CTL1-5 or OCTs to transport choline. Further investigations are extremely necessary to figure out this interesting and meaningful task to determine the choline transport pathway of tuft cells lacking CHT1.

### Mechanisms of ACh Synthesis in Tuft Cells

ChAT has been proven to be constitutively expressed in most tuft cells (Ting and von Moltke, 2019). Proskocil et al. examined ChAT exons in monkey lung and found that the form of ChAT expressed in bronchial epithelium was remarkably similar to ChAT expressed in neurons (Proskocil et al., 2004). Using electron microscopy, researchers have made it clear that the expressions of ChAT in mice localize in the cytosol as well as the nucleus (Kummer and Krasteva-Christ, 2014; Friedman et al., 2019). A recent study in intestinal epithelium has demonstrated that increased ChAT expression is related to the expansion of tuft cells following the increase of ACh secretion (Middelhoff et al., 2020).

Recently, it has been reported that the mitochondrial enzyme carnitine acetyltransferase (CarAT) contributes to ACh synthesis in peripheral tissues and non-neuronal cells (Wolf-Johnston et al., 2012; Kummer and Krasteva-Christ, 2014; Fujii et al., 2017). However, it is noteworthy that the synthesis of ACh in colon epithelial cells (not specifically mentioned tuft cells) is mainly catalyzed by ChAT rather than CarAT (Bader et al., 2014).

### Mechanisms of the Release of ACh in Tuft Cells

As discussed above, the biosynthesis of ACh occurs within the cytoplasm. Before participating in several pathophysiological processes, ACh has to be transported and released from cytoplasm. VAChT, encoded by *Slc18a3*, is involved in the progression of ACh release. VAChT helps to package ACh into vesicles, transports these vesicles to the plasma membrane and releases ACh via exocytosis (Friedman et al., 2019). In gastrointestinal and biliary tract (both in rodent and human), tuft cells lack the expression of VAChT and CHT1 (Schütz et al., 2015, 2019). This absence of VAChT suggests a new perspective in the ACh release of tuft cells. Several studies demonstrated that most epithelial cell types (with no specific mention on tuft cells) release ACh directly from the cytoplasm rather than concentrate it into vesicles. Furthermore, the expressions of OCT1 and OCT2, which are shown to possess ACh transport ability, have been identified in airway epithelial cells (Kummer and Krasteva-Christ, 2014). In another study in human placenta, OCT1 and OCT3, rather than OCT2, mediate the release of non-neuronal ACh (Wessler et al., 2001). It remains unknown whether tuft cells release ACh via OCTs and if they do, which subtype of OCTs participates in this process. This evidence may enlighten us about the different modes of VAChT-deficient tuft cells to release ACh: direct release, OCT-mediated way, or even the proteolipid mediatophore and gap junction (Kummer et al., 2008; Fujii et al., 2012; Schütz et al., 2015).

## Pathophysiological Roles of ACh Produced by Tuft Cells

### Regulation of Epithelial Homeostasis

Previous studies have found that tuft cell ablation would lead to impaired intestinal regeneration, suggesting the role of tuft cells in maintaining epithelial homeostasis (Westphalen et al., 2014; Middelhoff et al., 2020). An experiment on human intestinal organoid indicated that endogenous ACh increased cell division and differentiation to maintain the homeostasis of intestine (Takahashi et al., 2018; Brinkman et al., 2019). Tuft cells have been proven to be one of the two main sources of ACh within gastric and intestinal mucosa (Hayakawa et al., 2017). DCLK is a unique marker of the majority of tuft cells (Gerbe et al., 2011). In the intestinal epithelium, ACh regulates epithelial proliferation via muscarinic receptors. Muscarinic receptor blockage results in the expansion of DCLK1^+^ tuft cells and subsequently increases the synthesis and release of ACh in mouse model. These expanded tuft cells may also orchestrate with submucosal cholinergic nerve fibers to increase the secretion of ACh. Interestingly, mature tuft cells already existing in epithelium show no response to the reduced cholinergic signaling (Middelhoff et al., 2020). Lgr5 has been defined as a marker of intestinal stem cells (ISCs) with self-renewal ability (Barker et al., 2007). Lgr5^+^ ISCs are the cellular origins of goblet cells, Paneth cells, enterocytes, enteroendocrine cells and tuft cells in mice. Accumulating evidence indicates that ACh, especially non-neuronal ACh synthesized by epithelium (Takahashi et al., 2014), regulates Lgr5^+^ ISCs expansion via muscarinic acetylcholine receptor 3 (M3R) (Raufman et al., 2008). Ablation of DCLK1^+^ cells inhibits the proliferation of epithelium (Saqui-Salces et al., 2011). All these factors have confirmed the importance of tuft cells in intestinal epithelial homeostasis.

The interaction of ACh and M3R activates the expression of Wnt and induces canonical Wnt signaling (Labed et al., 2018). Wnt signaling has been reported to play a critical role in the self-renewal of intestinal epithelium (Karin and Clevers, 2016). As a co-activator to Wnt, Yes-associated protein (YAP) plays an important role in ACh-M3R axis as well (Hayakawa et al., 2017). Other signaling pathways promoting cell proliferation in human gastric cancer cells, such as epidermal growth factor receptor (EGFR) signaling and Akt pathway, have been demonstrated to be regulated by M3R signaling (Wang et al., 2016; Yu et al., 2017). However, it is hard to conclude that tuft cells would regulate cell proliferation in the same signaling pathway because of the lack of direct evidence.

The epithelial homeostasis regulatory function of tuft cells has also been supposed in lung growth in rhesus macaques and murine gastrointestinal tract development (Proskocil et al., 2004; Saqui-Salces et al., 2011). The increase of tuft cells after unilateral pneumonectomy suggests their role in rat pulmonary cell re-generation (Filippenko, 1978). In other studies, researchers established different gastric pathology mouse models and identified an expansion role of tuft cells under hyperplastic conditions such as injury and inflammation (Gerbe et al., 2011; Saqui-Salces et al., 2011).

Apart from ACh-M3R-Lgr5^+^ ISCs axis and others discussed above, tuft cells are capable of modulating epithelial homeostasis against helminth parasites via immune related pathways. It was found that tuft cell derived IL-25 could up-regulate group II innate lymphoid cells (ILC2s) to secret IL-13. The increase in IL-13 promotes goblet and tuft cell differentiation, hereby maintaining the integrity of murine mucosal barrier (Gerbe et al., 2016; von Moltke et al., 2016; Snelgrove and Lloyd, 2018; Miller et al., 2020). Another striking report discovered a novel context-specific function of tuft cells in pancreatitis. Long term chronic pancreatic injury resulted in the formation of tuft cells. These increased tuft cells highly expressed *Il25*, which indicated that tuft cells in murine pancreas responding to inflammatory in the same pathway to promote epithelial recovery (DelGiorno et al., 2020). Furthermore, recent studies showed that dietary and microbiota derived succinate may trigger tuft cells and type 2 immune response via succinate receptor1 (Sucnr1), indicating another mechanism for tuft cells in detecting invaders and maintaining epithelial homeostasis in mice (Lei et al., 2018; Schneider et al., 2018; McGinty et al., 2020). These immunomodulatory effects of tuft cells have been well discussed in other state-of-the-art reviews (Schneider et al., 2019; Ting and von Moltke, 2019).

However, it should be emphasized that the modulatory function of tuft cells in epithelial homeostasis should not be overestimated, for *in vivo* and *in vitro* studies showed DCLK1^+^ cells failed to form and maintain organoids (Westphalen et al., 2014). Therefore, further studies are needed to clarify the exact role of tuft cells in maintaining epithelial homeostasis.

### Regulation of Reflexes and Muscle Constriction

To figure out the regulatory effects of tuft cells in respiratory reflexes, Krasteva et al. (2011) established a mouse model which allowed the monitoring of respiratory events under administrating different substances in the upper cervical trachea. By inhaling various antagonists and agonists, researchers demonstrated that murine tracheal tuft cells were capable of sensing bitter substances and releasing ACh to activate adjacent vagal sensory nerve fibers, which subsequently resulted in respiratory reflexes (Krasteva et al., 2011). The evocation of respiratory reflexes reflected on the sharp changes in respiration combined with abrupt decreases in respiratory rate. Apart from the bitter substances, tuft cells are also capable of detecting bacterial products in airway lining fluid and thus conduct mucociliary clearance (Krasteva et al., 2012a; Hollenhorst et al., 2020; Perniss et al., 2020). Using *Avil^*cre*^:Chat^*fl/fl*^* mouse model which retained neural cholinergic signaling but conditional loss of ACh synthesis in tuft cells, Perniss et al. demonstrated that by detecting formylated bacterial peptides, tuft cells rather than cholinergic nerves released ACh activating mucociliary clearance (Perniss et al., 2020).

Similarly, tuft cells in murine urethra also exhibit the reflexes regulatory effect. In response to bitter, tuft cells release ACh and this ACh acts as a potential messenger passing signals on to vicinal cells or nerve fiber terminals with functional acetylcholine receptors. The activation of cholinergic receptors, dominantly nicotinic acetylcholine receptors (nAChR) α3 subunit, evokes sensory nerve fibers adjacent to urethral tuft cells penetrating into the urethral epithelium (Jung et al., 1999; Deckmann et al., 2014). The excitation of nerve fibers has been reported to function in micturition reflex and muscle constriction (Deckmann et al., 2014). However, a cholinergic negative autocrine feedback occurs in tuft cells (Deckmann et al., 2018). Urethral instillation of denatonium (25 mM; 50 μL) in rats drastically increased detrusor activity and this elevation could be antagonized by general nicotinic receptor blocker mecamylamine, indicating the role of tuft cells derived cholinergic signaling in urethra reflexes responding to taste transduction (Deckmann et al., 2014).

In addition to the activation on detrusor, ACh has also been reported as a well-accepted neurotransmitter inducing airway smooth muscle (ASM) constriction (Meurs et al., 2013). Among the five muscarinic receptors (M1-5R), M1R, M2R, and M3R have been determined in lungs. Although both of M2R and M3R have been proven to express in ASM, M2R is the predominant cholinergic receptor (Koarai and Ichinose, 2018). The stereotypical theory of ASM constriction is mediated by ACh released from cholinergic nerve terminals. However, non-neuronal ACh has recently been discovered to participate in the ASM constriction and even an accomplice in anaphylactic bronchoconstriction (Moffatt et al., 2004; Kummer and Krasteva-Christ, 2014; Kistemaker and Gosens, 2015). James and colleges found both epithelium removal or antagonist atropine abolished the constriction of ASM in mouse trachea induced by serotonin (Moffatt et al., 2004). They suggested that serotonin acted on 5-HT_2A_ receptors on mouse tracheal epithelial cells, resulting in the release of ACh from epithelial cells, leading to the constriction of ASM (Moffatt et al., 2004). In contrast, Kummer et al. (2006) did the similar experiment (epithelium removal and antagonist atropine treatment) in M2R and M3R-deficient mouse bronchi but found different results. Using videomorphometric studies, they found that precision-cut lung slices from M2R and M3R-deficient mouse maintained fully responsiveness to serotonin but lost responsiveness to cholinergic stimulation (Kummer et al., 2006). This evidence strongly suggested that other epithelium-derived factors rather than ACh mediated serotonin-induced ASM constriction. However, this discrepancy between two studies may due to two reasons. Firstly, atropine is a competitive antagonist to 5-HT_3_-receptor (Fan and Weight, 1994). The administration of high concentration of atropine inhibiting ASM constriction was a non-specific effect and the inhibitory effect of atropine did not indicate that serotonin-induced ASM constriction was in a muscarinic receptor-independent manner. Secondly, another nonnegligible reason is the different situations between trachea and bronchi that cholinergic chemosensory cells exist in the tracheal rather than bronchial mucosa (Krasteva et al., 2012a; Perniss et al., 2020). Epithelial cell has been identified a source of ACh in the airways, we should not exclude the possibility that epithelial cells-derived ACh participated in ASM constriction. Tuft cells have been found to situate mainly in trachea but rarely in bronchi in mice and they should not be underestimated in the regulation of ASM constriction. However, since current research focuses mostly on the epithelial ACh, rather than tuft cell-derived ACh in mice model, and the multiple lineages existing in airway epithelium, more works have to be done to make it clear whether tuft cells have unique roles in ACh-induced ASM constriction and whether the same function exists in human.

### Regulation of Airway Remodeling

In addition to the regulatory effects on epithelial homeostasis, non-neuronal ACh has also been proven to affect fibroblasts, myofibroblasts and inflammatory cells which contribute to airway remodeling in lung (Pieper et al., 2007). Administration of ACh promotes fibroblasts proliferation and differentiation into myofibroblast. Moreover, the activation of muscarinic receptors also enhances the proliferation of smooth muscle as well as fibroblasts (Meurs et al., 2013). Anti-muscarinic therapy is extremely effective in asthma-related airway remodeling (Gosens et al., 2005). In these studies, the source of ACh has not been well elucidated. However, a recent study investigated the role of DCLK1^+^ tuft cells in H1N1 influenza virus induced alveolar remodeling, in which Rane et al. (2019) observed an increase in DCLK1^+^ tuft cells in post-influenza murine lungs. These newly arisen tuft cells are adjacent to dysplastic epithelium in position and are derived from p63-expressing lineage-negative progenitors, the same as dysplastic epithelium (Rane et al., 2019). This is a previously unrecognized development of tuft cells, although the underlying mechanisms still need to be clarified. However, these findings imply the heterogenous origin of tuft cells and different pathological conditions may influence the differentiation and function of tuft cells.

### Participation in Inflammation

The potent immunomodulatory effects of tuft cells have already attracted plenty of attention. Haber et al. reported a single-cell survey of murine small intestinal epithelium and distinguished tuft cells into two mature subsets (Haber et al., 2017). Tuft-1 cells express neuron-related genes, while tuft-2 cells express immune-related genes. Both subsets express *Il25*, however, only tuft-2 cells express high levels of Th2 related cytokines, such as *Il4ra*, *Il13ral*, and *Il17rb* (Haber et al., 2017). Increasing lines of evidence focus on type 2 immune response mediated by tuft cells in helminth, protists, and virus infection (von Moltke et al., 2016; Noti, 2018; Wilen et al., 2018; Rane et al., 2019), whereas tuft cell derived ACh also contributes to inflammation. There are several reviews which contain in-depth knowledge involving the roles of tuft cells in immune responses (Snelgrove and Lloyd, 2018; Schneider et al., 2019; Ting and von Moltke, 2019). In the light of this rationale, we will only discuss the role of tuft cell derived ACh in inflammation.

ATP, as a danger signal, also has an impact on tuft cells. This regulation is mediated by ATP sensor P2Y2. The activation of tuft cells results in the generation of cysteinyl leukotriene, a mediator of inflammation, implying the function of murine tuft cells in inflammation (Ualiyeva et al., 2020).

As mentioned above, chemosensory is one of the main characteristics of tuft cells. Nasal tuft cells respond to the presence of bitter and bacterial quorum-sensing molecules via TRPM5 and gustducin, and seemed to be indispensable in trigeminal irritant-detection system (Tizzano et al., 2010). Activated by bitter compounds or bacterial metabolites, murine nasal tuft cells release ACh to activate nAChRs on contiguous peptidergic trigeminal nerve fibers. The evocation of trigeminal fibers causes substance P secretion, eventually evokes nerve fibers-related inflammation without the release of local inflammatory mediators. Substance P plays an important role in the process of neurogenic inflammation, which results in mast cell degranulation and plasma leakage from vessels (Saunders et al., 2014). The unique capacity of tuft cells, which release ACh to evoke nerve fibers mediating the inflammation, makes them the potential sentinels against hazardous substances in the environment.

### Promotion of the Initiation of Cancers

It has been reported that murine DCLK1^+^ tuft cells undergo a dramatic proliferation in response to chronic inflammation and act as a progenitor resulting in carcinogenesis (Saqui-Salces et al., 2011; Hayakawa et al., 2017). Westphalen and colleges generated *Dclk1-CreERT* BAC transgenic mice to label intestinal tuft cells. They determined a subset of DCLK1^+^ tuft cells with longevity and quiescence characteristics. These cells exhibit low expressions of proliferating cell nuclear antigen (PCNA) and Ki67, makers strictly associated with cell proliferation, and they can be labeled by BrdU for months (Itzkovitz et al., 2011; Westphalen et al., 2014). However, unless this rare subpopulation of DCLK1^+^ tuft cells encounter the loss of tumor suppressor gene combined with inflammation or injury challenges, will they overcome their intrinsic quiescence and become a potent tumor-initiation progenitor (Westphalen et al., 2014). Furthermore, Goto et al. (2019) found IL17RB, a tuft cell marker, is distinctively expressed by DCLK1^+^ mouse intestinal tumor cells and regulates the function of tuft cell-derived cancer stem cell. The expression of IL17RB was then proven to be a potential marker for the lineage tracing of human colorectal cancer stem cells (Goto et al., 2019). However, in human pancreas, DCLK1, a tuft cell marker in rodents, failed to co-localize with ChAT^+^ cholinergic tuft cells (Schütz et al., 2019). DCLK1^+^ epithelial cells in human pancreatic tissue need to be more rigorously reviewed and distinguished from cholinergic tuft cells.

Indeed, despite being described in intestinal and colorectal cancers, tuft cells have also been demonstrated to be a previously unrecognized cell of origin for a subset of human small cell lung cancer (SCLC) (Huang et al., 2018). Using CRISPR screen, Huang et al. validated that POU2F3 is an essential transcription factor in a subset of SCLC and POU2F3^+^ SCLC cells express the markers of tuft cell lineage, such as TRPM5, SOX9, CHAT, ASCL2, and AVIL. The underlying mechanism of SCLC initiation and whether cholinergic signaling participating in this initiation remain to be elucidated. Furthermore, tuft cells in pancreas have also been observed an appearance in response to tumor-initiating mutations, whereas they are absent in murine pancreas under normal conditions (Bailey et al., 2014).

In addition, it was found that tuft cells also modulate peritumoral neural microenvironment to promote carcinogenesis. In a gastric cancer mouse model, Hayakawa and colleges observed a significant increase of tuft cells in early stage, but a gradual loss of tuft cells accompanied by the increase of cholinergic innervation in the later progression of cancer (Hayakawa et al., 2017). ACh, derived from tuft cells and cholinergic nerves, stimulates gastric epithelium to up-regulate the expression of nerve growth factor (NGF), thus forming a positive feedback. This abnormal cholinergic signaling regulates Wnt and YAP pathways to promote the initiation of tumors. The shift in tuft cells and cholinergic innervation suggests that tuft cells, in part, interfere tumor initiation in the early stage (Hayakawa et al., 2017; Konishi et al., 2019).

Though tuft cells have been identified in the carcinogenesis, their role in tumor progression remains undetermined. In contrast with the tumor initiation state, tuft cells suffer a decline or absence in tumor progression (Bailey et al., 2014). It has been speculated that this reduction of tuft cells is probably attributed to the excessive growth of tumor cells. Unknown mechanisms inhibit tuft cell progenitors or even cause the loss of *DCLK1* expression (Middelhoff et al., 2017).

### Other Functions of Tuft Cells

Determined by RT-PCR, the mRNA expression of ChAT is significantly higher in taste tissue than in non-taste tissue, indicating the ACh synthesis capacity of taste buds. Among the three cell types of taste buds in mice, type II cells share the morphology and gene expression profiles with tuft cells (Sukumaran et al., 2017). In response to gustatory stimulation, type II taste cells synthesize and release ACh. This newly synthesized ACh in turn stimulates M3R on type II taste cells, inducing a disturbance of Ca^2+^ in these cells. The release of Ca^2+^ from internal stores enables continued ACh exocytosis, which forms an autocrine feedback and potentiates taste-evoked signals. Besides the enhancement in ACh production, the ACh also increases the release of taste neurotransmitter ATP. Taste-evoked ATP secretion was detected under different conditions with or without muscarinic receptor blockage. Disturbance in cholinergic receptors results in a decrease in ATP secretion (Dando and Roper, 2012). Surprisingly, besides type II taste cells, there is no other study focusing on the ATP regulatory effect of tuft cells.

## Potential Clinical Application

With increasing evidence highlighting the role of ACh as an autocrine and paracrine hormone, cholinergic targets have drawn widespread attention in pulmonary diseases (Meurs et al., 2013; Voisin et al., 2017), Hirschsprung’s disease (O’Donnell et al., 2020) and cancers (Westphalen et al., 2014; Schütz et al., 2015; Pozo et al., 2018; Friedman et al., 2019; Goto et al., 2019). Tuft cells, considered as a crucial non-neuronal ACh source, have also been discussed in several disease models.

### Cancer

As demonstrated above, tuft cell is not only the progenitor during carcinogenesis, but also a potent assistant modulating tumor-initiating microenvironment. Due to this reason, tuft cells become potential targets for tumor prevention and treatment.

Given that ACh-NGF-YAP axis promotes gastric tumorigenesis, inhibition of ACh, M3R, NGF is considered to be a potent therapeutic strategy against gastric cancer. A preclinical study showed that disturbance in ACh-NGF axis with anti-NGF antibody or downstream Trk inhibitors suppressed stomach cancer (Hayakawa et al., 2017). Furthermore, the inhibition of ACh synthesis with ChAT antagonist BW813U (2.5 mg/kg, thrice a week, i.p.) also suppressed the lung tumor formation in immunodeficient mice (Friedman et al., 2019). However, although ChAT antagonist showed potent antitumor effects in mice, it has not been tested in human cancers. The anti-tumor effects of M3R antagonist has been well established in multiple cancers, such as gastrointestinal cancers and lung cancers (Friedman et al., 2019; Konishi et al., 2019).

Using CRISPR-Cas9 technology combined with organoid culture, lineage-tracing becomes possible in human cancers. However, long-term marker targeting, Lrg5 for example, gives rise to inevitable liver toxicity (Tian et al., 2011). Considering IL17RB is amenable in marking tuft cell-like cancer stem cells with no expression on normal stem cells, IL17RB may be a potential marker for long-term targeting. Goto et al. have already identified a subpopulation of human colorectal cancer with IL17RB tuft cell-like cancer cells in human biopsy samples (Goto et al., 2019).

### Hirschsprung’s Disease and Other Intestinal Disorder

A recent study on Hischsprung’s disease (HSCR) suggested a new role of tuft cells (O’Donnell et al., 2020). Using immunolabeling and qRT-PCR analysis, O’Donnell et al. (2020) observed a decrease of DCLK1^+^ tuft cells in human HSCR tissue specimens. The authors supposed that the reduction of tuft cells is, in part, responsible for the bowel dysmotility. However, lacking supporting experimental evidence, whether tuft cells could be used as a marker to distinguish normal intestine from unbalanced homeostasis remains to be elucidated. Available data are insufficient to postulate such a conclusion.

Previous studies implicated that ACh metabolism altered in rectosigmoid colon from HSCR patients and other intestinal infection and inflammation (Hardy et al., 1993; Hirota and McKay, 2006; Takahashi, 2020). It is necessary to find ways to interfere with the abnormal cholinergic system to maintain the intestinal homeostasis. ACh has been proven a pivotal role in regulating enteric epithelial ion transport (Hirota and McKay, 2006). However, at present, there is no direct evidence to prove that tuft cell-derived ACh participates in epithelial ion transport.

### Pulmonary Diseases

ACh has been proven to be involved in the bronchoconstriction, airway remodeling and allergic inflammation. The sources of ACh in the lung are intricate that both parasympathetic nerves and other non-neuronal cells such as tuft cells can contribute to the release of ACh. *Trpm5*-deficient mice that lack tuft cells are more susceptible to infection, suggesting that tuft cells participate in airway anti-infections (Perniss et al., 2020).

Tiotropium, a long-lasting non-specific muscarinic antagonist, significantly inhibited the accumulation of eosinophils and Th2 inflammation, and antagonized the proliferation of fibroblasts and myofibroblasts, thus eliminating the airway remodeling in asthma and chronic obstructive pulmonary disease (COPD) (Bos et al., 2007; Pieper et al., 2007). In sputum from patients with COPD, formylated bacterial peptides have been detected and tuft cells have been reported to sense virulence-associated formyl peptides and release ACh to activate tracheal mucociliary clearance (Perniss et al., 2020). In another study of COPD, the authors found that tiotropium could reduce IL-17A induced CXCL8 release and eliminate inflammation, indicating the potential of anti-cholinergic therapy in controlling COPD (Meurs et al., 2013). However, vagotomy of parasympathetic nerve in the lungs effectively inhibited the inflammation and bronchial hyperresponsiveness in dogs, indicating the dominate role of nerves, rather than non-neuronal cells (Liu et al., 2014; Voisin et al., 2017).

Although tuft cells do not show the uppermost role in symptomatic relief of asthma, they are crucial in taste transduction, and specific anti-tuft cells derived ACh application may benefit asthma patients suffering side effects from bronchodilators. Atropine derivatives are widely used as inhaled bronchodilators. However, unpleasant taste is the unavoidable side effect of this application (Newnham, 2001). Given that type II taste cells play the pivotal roles in murine taste transduction cascade in ACh-M3R-dependent manner (Dando and Roper, 2012), specific blockage of type II taste cells might reduce the sensitivity of taste signaling and increase tolerance to cholinergic drugs.

### Overactive Bladder

Antimuscarinic drugs have been applied to the treatment of overactive bladder (Andersson, 2016). Overactive bladder syndrome and detrusor overactivity are the main symptoms of overactive bladder. As demonstrated above, responding to taste transduction, tuft cells derived cholinergic signaling regulates urethra reflexes and detrusor constriction. In an experiment on urethane-anesthetized rats, urethral instillation of denatonium (25 mM; 50 μL) drastically increased detrusor activity and this elevation could be antagonized by general nicotinic receptor blocker mecamylamine (Deckmann et al., 2014). In an analysis including 31 women with overactive bladder syndrome, higher urinary ACh was found in anticholinergic therapy responders compared to nonresponders after 12-week anticholinergic medications treatment (Sheyn et al., 2019). Thus, pharmacological applications to overactive bladder should be advanced and supported by more selective compounds. Since tuft cells serve as a source of ACh in overactive bladder, specific targeting on tuft cell-derived ACh may be a prospective solution.

## Conclusion

Tuft cells are rare solitary chemosensory cells distributed in mucosal epithelium throughout mammalian organs. The physiological roles include chemosensing, immunomodulation, and ACh synthesis. In this review, the main pathophysiological roles of tuft cell derived ACh are summarized and discussed. Tuft cell derived ACh participates in maintaining epithelial homeostasis, modulating airway remodeling, regulating respiratory reflex, promoting inflammation, initiating carcinogenesis, and in producing ATP and so on.

Increasing single cell surveys in epithelium unravel the biological characteristics of tuft cells. However, there remain questions and conflicts. Although it is believed that there is barely no tuft cell existing in lower airway of mice, a tuft cell-like variant of human SCLC was found (Huang et al., 2018). Therefore, the distribution of tuft cells in human lung still needs to be carefully investigated. The knockout of *Pou2f3* gene indicates the specific deletion of tuft cells and *Pou2f3*^–/–^ mice exhibit impaired mucosal type 2 responses and epithelial regeneration (Gerbe et al., 2016; Middelhoff et al., 2020). In this respect, the long term targeting on IL17RB to monitor tuft cell-like human colorectal cancer still needs further research to avoid the potential damage to the homeostasis of colorectal mucosa. In the condition of tasting bitter, tuft cells have been proven to evoke urethral reflexes via the release of ACh. However, this evocation can be mainly but not entirely abrogated by the local administration of general nicotinic receptor blocker (Deckmann et al., 2014). This deviation may due to the additional involvement of muscarinic acetylcholine receptors, and other functional co-transmitters or the insufficient local administration of nAChR blocker. Finding ways for a more effective administration method may be the leading issue for further development of research models and clinical applications.

As a major characteristic of tuft cells, the regulation of ChAT expression and functions of tuft cell derived ACh remain intricate. Furthermore, there are multiple types of cells and nerve terminals in the context of tuft cells, therefore, more works need to be done to unravel the crosstalk between the tuft cells and the adjacent cells as well as nerve terminals.

## Author Contributions

JP conducted the systematic literature search and wrote the manuscript. LZ contributed to the literature search and manuscript. XS helped to edit the manuscript. JH put forward the idea of the manuscript and helped with editing. All authors contributed to the article and approved the submitted version.

## Conflict of Interest

The authors declare that the research was conducted in the absence of any commercial or financial relationships that could be construed as a potential conflict of interest.
